# Bone mineral density at the hip and its relation to fat mass and lean mass in adolescents: the Tromsø Study, Fit Futures

**DOI:** 10.1186/s12891-018-1933-x

**Published:** 2018-01-19

**Authors:** Anne Winther, Lone Jørgensen, Luai Awad Ahmed, Tore Christoffersen, Anne-Sofie Furberg, Guri Grimnes, Rolf Jorde, Ole Andreas Nilsen, Elaine Dennison, Nina Emaus

**Affiliations:** 1 Division of Neurosciences, Orthopedics and Rehabilitation Services, University Hospital of North Norway, Tromsø, Norway; 2 Department of Health and Care Sciences, UiT The Arctic University of Norway, Tromsø, Norway; 3 Institute of Public Health, College of Medicine and Health Sciences, United Arab Emirates University, Al Ain, United Arab Emirates; 4 Finnmark Hospital Trust, Alta, Norway; 5 Department of Community Medicine, UiT The Arctic University of Norway, Tromsø, Norway; 6 Division of Internal Medicine, University Hospital of North Norway, Tromsø, Norway; 7 Tromsø Endocrine Research Group, Department of Clinical Medicine, UiT The Arctic University of Norway, Tromsø, Norway; 8 MRC Lifecourse Epidemiology Unit, Southampton, UK; 9 Victoria University, Wellington, New Zealand

**Keywords:** aBMD, Fat mass, Lean mass, DXA, Adolescents, Population-based study

## Abstract

**Background:**

Positive association between body weight and bone mass is well established, and the concept of body mass index (BMI) is associated with higher areal bone mineral density (aBMD) and reduced fracture risk*.* BMI, that comprises both fat mass (FM) and lean mass (LM) may contribute to peak bone mass achievement in different ways. This study explored the influence of body composition in terms of total body LM and FM on hip aBMD-values in adolescence.

**Methods:**

In 2010/2011, 93% of the region’s first-year upper-secondary school students (15–17 years old) in Tromsø, Norway attended the Tromsø Study, Fit Futures. Areal BMD at femoral neck (aBMD_FN_) and total hip (aBMD_TH_) (g/cm^2^), total body LM and FM (g) were measured by dual energy X-ray absorptiometry (DXA). Height and weight were measured, and BMI calculated. Lifestyle variables were collected by self-administered questionnaires and interviews, including questions on time spent on leisure time physical activity. Stratified analyses of covariance and regression models included 395 girls and 363 boys. Crude results were adjusted for age, height, sexual maturation, physical activity levels, vitamin D levels, calcium intake, alcohol consumption and smoking habits.

**Results:**

Unadjusted distribution indicated higher aBMD-levels at higher LM-levels in both genders (*p* < 0.001), but higher aBMD at higher FM-levels were found only in girls (*p* < 0.018). After multiple adjustments, aBMD_FN_-levels in girls were associated by 0.053 g/cm^2^ and 0.032 g/cm^2^ per standard deviation (SD) change in LM and FM (*p* < 0.001). Corresponding values in boys were 0.072 and 0.025 (*p* < 0.001). The high LM groups accounted for the highest aBMD-levels, while aBMD-levels at the LM/FM-combinations indicated different patterns in girls compared to boys. The adjusted odds ratio (95% CI) for low levels of aBMD_FN_ was 6.6 (3.4,13.0) in boys, compared to 2.8 (1.6,4.9) in girls per SD lower LM.

**Conclusions:**

LM and FM should be regarded as strong predictors for bone mass and hence bone strength in adolescents*.* A gender specific difference indicated that high lean mass is of crucial importance prominently in boys. In adolescents with low lean mass, especially in girls, high fat mass may partially ameliorate the effect of deficient lean mass levels.

## Background

Osteoporoses as well as osteoporotic fractures are major health problems in Western Societies, and areal bone mineral density (aBMD) is a strong predictor of future fracture risk [1]. An individual’s aBMD-level in the elderly is a result between peak bone mass (PBM) achieved during growth and subsequent bone loss [2]. The massive skeletal changes during adolescence, especially through puberty onset and the following growth spurt are characterized by rapid modelling and remodelling [3]. This makes the adolescent period important for an individual’s PBM level as basis for the following inevitable bone loss in later life [3, 4]. The positive association between body weight and bone mass is well established, and body weight adjusted for stature is the largest single determinant of bone mass variability in adults [5]. Therefore body mass index (BMI) is positively associated with bone mass, and weight stability or weight maintenance in adults as well as in youths, is regarded protective against future fracture risk [6–9].

In a previous study of an adolescent population, association between BMI and aBMD levels at the hip were modest, but statistically significant [10]. However, there are studies suggesting that the positive association between BMI and bone mineral parameters is limited below a certain BMI threshold [11, 12].

The concept of BMI comprises stature and weight, while body weight is a compound of bone, fat and muscle. Lean mass (LM) is likely to be responsible for the positive association between BMI and bone mineral parameters [11, 13–16], whereas the role of fat mass (FM) related to PBM is not so clear [14, 17]. The last position statement from National Osteoporosis Foundation on PBM development concludes with consensus about LM’s positive effect on bone in a younger population, while the effect of FM on bone accretion is still under debate [18]. According to Farr and Dimitri [19] methodological problems may contribute to the diverging understanding of FM’s impact on bone, and they state that a linear relationship between FM and bone mass seems unlikely. They suggest both advantages and disadvantages of FM, probably because of changes in hormonal milieu, including an age and gender dependent fat-bone relationship.

In a former study we found that higher BMI-levels were related with lower levels of physical activity (statistically significant in boys) [20]. Surprisingly, further exploration showed a gender dependent association between physical activity and aBMD. Sedentary behaviour was negatively associated with aBMD in boys only, whereas in girls there was a positive association between lower physical activity levels and aBMD. Furthermore, this pattern still persisted two years later [20]. As both physical activity and FM is correlated with muscle mass [7], physical activity may be important in dissecting out the relationship between body composition and bone health.

On this basis we wanted to study the influence of body compositions measures on aBMD at the hip in a large representative sample of Norwegian adolescents, homogenous in age and ethnicity. We hypothesized that the relationship between fat mass and lean mass and areal bone mineral density would vary between girls and boys. We furthermore aimed to examine whether there are certain gender specific combinations of total body lean mass and fat mass, which are more beneficial for bone health.

## Methods

### Study population and design: Fit Futures

The Tromsø Study [21] is a population-based study with repeated health surveys in the municipality of Tromsø, Norway, inviting all residents in specific age groups. The Fit Futures (TFF) inviting a youth cohort is an expansion of the Tromsø Study, performed in collaboration with the University Hospital of North Norway (UNN HF), UiT The Arctic University of Norway and the Norwegian Institute of Public Health.

**Fig. 1:**
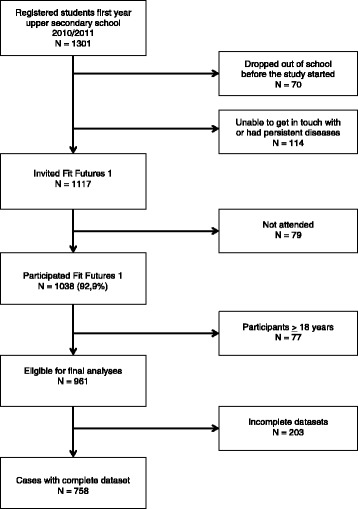
Flowchart of participation in The Tromsø Study, Fit Futures 1 (2010–2011)

In 2010/2011 all first-year upper-secondary school students in the two neighbouring municipalities Tromsø and Balsfjord were invited to participate in the first cross sectional study TFF1 [10], a multipurpose health study. The invited cohort included 1117 participants, of which 1038 adolescents (530 boys) attended the survey providing an attendance rate of 92.9%. All together the cohort consisted of 961 participants younger than 18. Participants with any missing values in the variables included in the final models were excluded, and this paper consists of 395 girls and 363 boys with complete data sets (Fig. 1).

Information about the study was given in classrooms and written information was distributed through the schools web sites. Participants signed a declaration when arriving at the study site, in addition participants younger than 16 had to bring written permission from their guardians. Dedicated research technicians performed the examinations in a well-established research unit at UNN HF. The Norwegian Data Protection Authority (reference number 2009/1282) and The Regional Committee of Medical and Health Research Ethics (2011/1702/REK Nord) approved the study in July 2010 and October 2011, respectively.

### Measurements

The main outcomes in the present study were areal bone mineral density at femoral neck (aBMD_FN_) and at total hip (aBMD_TH_) expressed as g/cm^2^, measured by dual x-ray absorptiometry (DXA) (GE Lunar prodigy, Lunar Corporation, Madison, WI, USA), and analysed with enCORE paediatric software [22]. The software used is the Lunar paediatric application, and comprise reference values for healthy children and youth 5 to 19 years old. From whole body scans the software also gave information on FM and LM in grams, which we converted into kilograms. At arrival, information on pregnancy was obtained through a clinical interview. In cases of possible pregnancy, participants were excluded from DXA scanning. Altogether 11 of the 1038 DXA scans were lost, mainly because of poor quality. In vivo, the densitometer coefficient of variation in percentage (% CV = (SD)/mean × 100) has been estimated to 1.17 and 1.72% for aBMD_TH_ and aBMD_FN_ respectively [23].

Height and weight were measured in all participants to the nearest 0.1 cm and 0.1 kg on an automatic electronic scale, the Jenix DS 102 stadiometer (Dong Sahn Jenix, Seoul, Korea). Measurements were performed according to standardized procedures in the Tromsø Study, with participants wearing light clothing without shoes. BMI was calculated as weight (kg) divided by the height squared (m^2^). Non-fasting blood samples were collected, and analysed for serum 25 hydroxy vitamin D levels, with a reference value of 50–113 nmol/l, and a CV < 6% [24].

### Questionnaires

Using the “Saltin-Grimby” 4 scale question [25], participants rated their time spent on physical activity an average week during the last year. They graded their physical activity as (1) sedentary activities only, (2) moderate activity like walking, cycling or exercise at least 4 h a week, (3) participation in recreational sports at least 4 h a week or (4) hard training and sports at a competitive level several times of week. Further on these four levels are denoted as “Sedentary”, “Moderate”, “Sports” and “Hard training”.

Sexual maturation was based on girls’ self reported menarche age and boys’ rating at the Puberty Development Scale (PDS) [26]. The boys rated four secondary sexual characteristics on a scale ranging from (1) not yet started to (4) complete. The PDS-score was calculated as mean score of the four items. Collection of these variables along with smoking habits, alcohol consumption, ethnicity, diseases and use of hormonal contraceptives and medications are described in detail elsewhere [10]. Shortly, those reported “never drinking alcohol” were compared to those reporting “sometimes”, ever smokers were compared to never smokers. As calcium intake in Nordic populations in general is sufficient [27], we estimated calcium consumption separating low diary consumers, with potential inadequate calcium levels, from the rest. Based on five questions about amount and frequency of cheese and diary drinks consumption, we dichotomized the calcium consumption into “Low”; not eating cheese once a week or not having diary drinks daily, and “Sufficient” when having cheese weekly or diary drinks daily. Diseases (ICD_10_) like hypothyroidism (E03), diabetes type 1(E10), eating disorders (F50.9), celiac disease (K90.0) and arthritis (M13) were separated from other diseases. Just like medication (ATC); plain corticosteroids (D07A), thyroid preparations (H03A), antiepileptic (N03A), corticosteroids (R01AD), glucocorticoids (inhalants) (R03BA) and corticosteroid for systemic use (H02A), were separated from other medications.

### Statistics

All analyses were performed sex stratified, as magnitude and tempo of bone mass acquisition differs between girls and boys [28]. We calculated means and SD for the continuous variables; age, height, weight, BMI, FM, LM, aBMD_FN,_ aBMD_TH_, serum vitamin D levels and puberty score. Percentage and numbers described physical activity levels, smoking habits, and calcium intake and alcohol consumption. Correlation between body composition and anthropometric measurements were explored. As body weight was highly correlated to BMI and FM we excluded weight from further analyses (Pearson’s correlation coefficient > 0.7). Mean values of body composition at the four physical activity levels were compared, and trends estimated by linear regression.

**Fig. 2:**
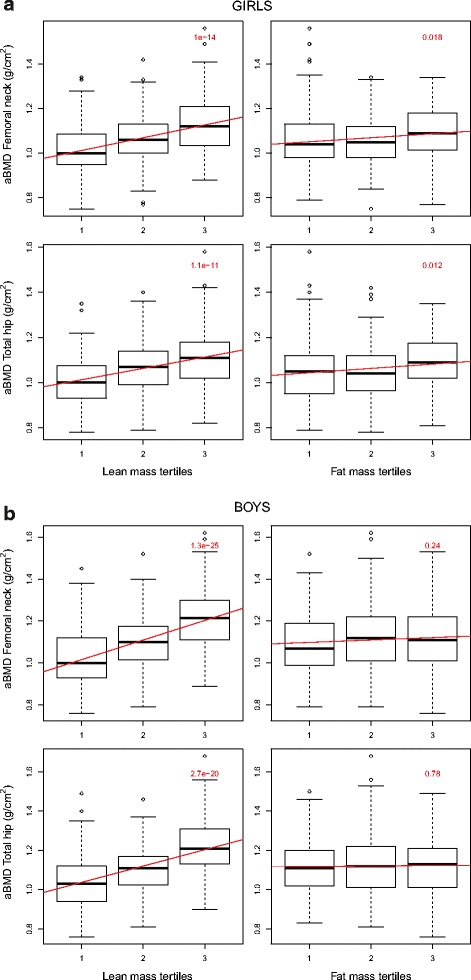
**a** Distribution of aBMD at the hip across lean mass and fat mass tertiles separately (crude values), including trend lines and p-values, for girls 15–17 years old. The Tromsø Study, Fit Futures 1. LM tertile 1; ≤ 36.75 kg, LM tertile 2; 36.76–40.31 kg, LM tertile 3; ≥ 40.32 kg: FM tertile 1; ≤ 15.48 kg, FM tertile 2; 15.49–21.01 kg, FM tertile 3; ≥ 21.02 kg. **b** Distribution of aBMD at the hip across fat mass and lean mass tertiles separately (crude values), including trend lines and *p*-values, for boys 15–17 years old. The Tromsø Study, Fit Futures 1. LM tertile 1; ≤ 50.88 kg, LM tertile 2; 50.89–56.41 kg, LM tertile 3; ≥ 56.42 kg; FM tertile 1; ≤ 8.04 kg, FM tertile 2; 8.05–15.29 kg, FM tertile 3; ≥ 15.30 kg

We explored the distribution of aBMD across FM and LM tertiles and estimated trends by linear regression (Fig. 2). In girls the tertiles was defined by cut-off as following; FM low ≤ 15.48 kg, FM medium 15.49–21.01 kg and FM high ≥ 21.02 kg, subsequently LM low ≤ 36.75 kg, LM medium 36.76–40.31 kg and LM high ≥ 40.32 kg. In boys the cut-offs were set to FM low ≤ 8.04 kg, FM medium 8.05–15.29 kg and FM high ≥ 15.30 kg, corresponding cut-offs for LM was low ≤ 50.88 kg, LM medium 50.89–56.41 kg, LM high ≥ 56.42 kg.

When we explored the associations of BMI, LM and FM with aBMD, there was a statistical significant interaction between LM and FM. According to Kirkwood and Stern [29] it is not adequate to report the effect of an exposure on an outcome controlled for a given confounder, when interaction exists. Instead separate exposure effects for each stratum of the confounder should be reported [29]. As there are no agreed cut-offs for FM or LM, we stratified the multiple regression analyses according to tertiles of these variables, as described above (Table 3). To simplify the interpretation, units for FM and LM variables were transformed from kilo to SD based on the study population’s mean levels for these variables, in girls and boys respectively.

**Fig. 3:**
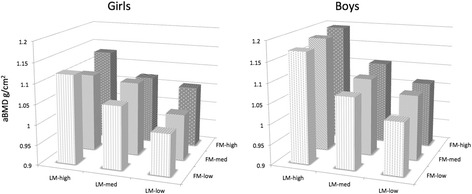
Femoral neck aBMD displayed as mean (g/cm^2^) at different lean mass-fat mass combinations for girls and boys15–17 years old. The Tromsø Study, Fit Futures 1. Each bar represent a sample size of 29 or more individuals, and all estimates are adjusted for age, height, sexual maturation, physical activity levels, calcium intake, vitamin D levels, alcohol consumption and smoking habits. Girls: FM low; ≤ 15.48 kg, FM med; 15.49–21.01 kg, FM high; ≥ 21.02 kg; LM low; ≤ 36.75 kg, LM med; 36.76–40.31 kg, LM high; ≥ 40.32 kg. Boys: FM low; ≤ 8.04 kg, FM med; 8.05–15.29 kg, FM high; ≥ 15.30 kg. LM low; ≤ 50.88 kg, LM med; 50.89–56.41 kg, LM high; ≥ 56.42 kg

By one-way between-group analyses of covariance (ANCOVA) we compared means and confidence intervals (CI) for aBMD across FM and LM tertiles in simple as well as in more complex models adjusted for age, height, sexual maturation, physical activity levels, calcium intake, vitamin D levels, alcohol consumption and smoking habits. The trends were estimated by linear regression, and by 2-ways ANCOVA (Fig. 3) we compared aBMD-values at different LM/FM-combinations. Diseases, medications or contraceptives known for influencing bone acquisition, were statistical non-significantly associated with aBMD in preliminary analyses. Participants reporting such events were therefore included in the analyses. And to keep the models simple, these confounders were not included in our models.

At the end we estimated by logistic regression, the impact of lower LM and FM levels together with the above described covariates, on the likelihood of having an aBMD_FN_-score ≥ 1 SD below mean versus not. This cut-off value was based on the hypothesis of a fracture risk reduction by 50%, when the bone mass amount is raised by one standard deviation at the end of skeletal maturation [4]. We checked assumptions of normality, linearity, homogeneity of variances and homogeneity of regression slope, without finding violations. Normality plots and test (Kolmogorov-Smirnov n > =50) and homogeneity of residuals (Levene’s test) were all found satisfactory.

All analyses were performed by Statistical Package of Social Sciences software (SPSS v. 22) and values of *p* < 0.05 were considered significant.

## Results

Complete data sets were available for 395 girls and 363 boys aged 15–17 years old i.e. 68% of the eligible population, 73% of the participating youths (Fig. 1), and their characteristics are displayed in Table 1.

**Table 1:** Characteristics for participants 15–17 years old

	GIRLS *n* = 395	BOYS *n* = 363
Age, year	16.6 (0.4)	16.7 (0.4)
Height, m	1.65 (0.06)	1.77 (0.07)
Weight, kg	60.7 (11.5)	69.8 (13.3)
BMI, kg/m^2^	22.2 (3.9)	22.2 (3.8)
Lean mass, kg	38.7 (4.6)	53.9 (6.6)
Fat mass, kg	20.1 (8.8)	14.1 (10.1)
Menarche age girls, years	13.0 (1.2)	
Puberty boys, PDS score (Range 1; Not started – 4; Completed)		3.3 (0.4)
Puberty boys, PDS groups, % (n) Completed/ Underway/ Barely started/ Not started		8.3 (30)/75.2 (273)/16.5(60)/0(0)
Serum VitD, nmol/l	54.8 (23.1)	40.8 (20.2)
Physical activity levels, % (n)		
• Sedentary	12.7 (50)	27.8 (101)
• Moderate	40.0 (158)	23.7 (86)
• Sports	30.1 (119)	22.0 (80)
• Hard training	17.2 (68)	26.4 (96)
Smoking habits, daily or sometimes, % (n)	19.2 (76)	21.2 (77)
Alcohol consumption, sometimes, % (n)	74.9 (296)	68.0 (247)
Calcium intake, cheese weekly/diary daily, % (n)	88.4 (349)	90.4 (328)
aBMD, g/cm^2^		
• Total hip	1.062 (0.123)	1.120 (0.149)
• Femoral neck	1.069 (0.124)	1.109 (0.149)

Displayed as mean (SD) for continuous variables and % (n) for categorical variables. The Tromsø Study, Fit Futures 1

The waist majority (98%) of the cohort reported white ethnicity. Among girls, 123 (31%) reported use of oral contraceptives. A total of 10 (1,3%) and 18 (2,3%) participants reported diseases and medication known to affect bone, respectively (data not shown). There were no statistically significant relationships between overall BMI and physical activity levels, whereas higher levels of physical activity were significantly associated with higher levels of LM and lower levels of FM (*p* ≤ 0.001) (data not shown).

Distribution of crude aBMD-levels at the hip sites indicated trends of higher median values across LM-tertiles in both genders (*p* < 0.001), whereas across FM-tertiles such trends were seen in girls only (*p* ≤ 0.018) (Fig. 2a and b). The ANCOVA showed largely the same patterns after multiple adjustments. For girls, there were trends of higher aBMD-levels at high LM (*p* < 0.001) and FM tertiles (*p* ≤ 0.016) at femoral neck and total hip. In boys there were positive trends across LM tertiles at both femoral sites (*p* < 0.001). However, across FM tertiles a weak, but significant positive trend (*p* = 0.010) was found at the femoral neck site, but not at total hip (data not shown).

Simple linear regression analyses (Table 2) showed positive associations between LM and aBMD, with beta values of 0.054 g/cm^2^ in girls and 0.083 g/cm^2^ in boys (*p* < 0.001) at femoral neck per SD higher LM. Correspondingly per SD higher FM; 0.028 g/cm^2^ (*p* < 0.001) and 0.011 g/cm^2^ (*p* = 0.15) for girls and boys respectively. In multiple regression models, LM and FM were significantly related to aBMD along with a statistically significant LM*FM interaction term, in girls minus 0.011 (*p* = 0.006) and in boys minus 0.016 (*p* = 0.005) (data not shown).

**Table 2:** Lean mass’ and fat mass’ associations with hip aBMD (g/cm^2^) given per SD change in body composition, for boys and girls 15–17 years old. The Tromsø Study, Fit Futures 1

	GIRLS						BOYS					
	Unadjusted			Adjusted^a^			Unadjusted			Adjusted^a^		
	B	*p*	*R*^*2*^ (%)	B	*p*	*R*^*2*^ (%)	B	*p*	*R*^*2*^ (%)	B	*p*	*R*^*2*^ (%)
Femoral neck												
BMI	0.036	< 0.001	9.2				0.037	< 0.001	6.4			
Lean mass	0.054	< 0.001	18.8	0.053	< 0.001	26.8	0.083	< 0.001	30.8	0.072	< 0.001	39.7
Fat mass	0.028	< 0.001	5.2	0.032	< 0.001	22.7	0.011	0.146	0.6	0.025	< 0.001	31.1
Total hip												
BMI	0.040	< 0.001	11.5				0.038	< 0.001	6.7			
Lean mass	0.048	< 0.001	15.4	0.057	< 0.001	25.2	0.077	< 0.001	26.6	0.077	< 0.001	39.1
Fat mass	0.030	< 0.001	5.9	0.036	< 0.001	21.0	0.009	0.228	0.4	0.026	< 0.001	28.9

^a^Adjusted for age, height, sexual maturity, physical activity, calcium intake, vitamin D levels, alcohol consumption and smoking habits

The regression analyses of lean mass is not adjusted for fat mass, and vice versa

Furthermore, in girls LM was significantly associated with aBMD_FN_ in all FM groups, with the highest increment per SD change, in the lowest FM group (beta = 0.087 g/cm^2^, *p* < 0.001). Correspondingly FM was significantly associated with aBMD_FN_ in the adjusted models, for all LM groups (*p* ≤ 0.017), with the highest beta (0.038 g/cm^2^, *p* = 0.009) seen in low LM group (Table 3). By contrast in boys, LM was positively associated with aBMD_FN_ in all FM groups (*p* < 0.001), with highest beta value in the medium tertile (0.095 g/cm^2^), while FM only had significant relationship with aBMD in the low LM group (beta = 0.040 g/cm^2^, *p* = 0.010). For both genders analyses at the total hip site revealed similar patterns, suggesting a consistent association between LM and FM and femoral aBMD.

**Table 3:** Relationships between hip aBMD and lean mass stratified by fat mass tertiles, correspondingly; fat mass stratified by lean mass tertiles, given per SD change and displayed as g/cm^2^. For girls and boys 15–17 years of age. The Tromsø Study, Fit Futures 1

			GIRLS *n* = 395				BOYS *n* = 363				
			Unadjusted		Adjusted^a^		Unadjusted			Adjusted^a^	
		n	Beta	*p*	Beta	*p*	n	Beta	*p*	Beta	*p*
Femoral neck											
Lean Mass (SD)	FM - Low	132	0.069	< 0.001	0.087	< 0.001	121	0.080	< 0.001	0.073	< 0.001
	FM - Medium	132	0.061	< 0.001	0.049	0.002	121	0.102	< 0.001	0.095	< 0.001
	FM - High	131	0.036	< 0.001	0.035	0.002	121	0.067	< 0.001	0.063	< 0.001
Fat Mass (SD)	LM - Low	132	0.040	0.006	0.038	0.009	121	0.019	0.215	0.040	0.010
	LM - Medium	132	0.022	0.067	0.030	0.017	120	0.006	0.633	0.017	0.180
	LM - High	131	0.012	0.144	0.024	0.009	122	−0.014	0.154	0.003	0.776
Total hip											
Lean Mass (SD)	FM - Low	132	0.062	< 0.001	0.088	< 0.001	121	0.074	< 0.001	0.065	< 0.001
	LM - Medium	132	0.061	< 0.001	0.056	< 0.001	121	0.096	< 0.001	0.105	< 0.001
	FM - High	131	0.025	0.008	0.034	0.003	121	0.063	< 0.001	0.069	< 0.001
Fat Mass (SD)	LM - Low	132	0.045	0.002	0.045	0.002	121	0.018	0.268	0.041	0.009
	LM - Medium	132	0.023	0.052	0.026	0.032	120	−0.004	0.734	0.008	0.516
	LM - High	131	0.016	0.065	0.030	0.001	122	−0.009	0.360	0.010	0.366

^a^Adjusted for age, height, sexual maturity, physical activity levels, calcium intake, vitamin D levels, alcohol consumption and smoking habits

Girls: FM-Low; ≤ 15.48 kg, FM-Medium; 15.49–21.01 kg, FM-High; ≥ 21.02 kg

LM-Low; ≤ 36.75 kg, LM-Medium; 36.76–40.31 kg, LM-High; ≥ 40.32 kg

Boys: FM-Low; ≤ 8.04 kg, FM-Medium; 8.05–15.29 kg, FM-High; ≥ 15.30 kg

LM-Low; ≤ 50.88 kg, LM-Medium; 50.89–56.41 kg, LM-High; ≥ 56.42 kg

Higher aBMD-values at femoral neck across LM/FM-combinations in boys were contrasted by the more complex patterns seen in girls (Fig. 3). The high LM groups accounted for the highest aBMD-levels in both genders, but especially the girls in the low LM/high FM-combination had higher mean aBMD, though not statistically significant, compared to their slimmer peers.

The odd ratio (OR) for having aBMD score one SD below mean values, also suggested sexual dimorphism; for each SD lower LM the OR raised by 6.6 (95% CI: 3.4–13.0) in boys and 2.8 (95% CI: 1.6–4.9) in girls after multiple adjustments (data not shown). Similar analyses for FM turned out statistically non significant.

## Discussion

### Summary

We found a gender specific variation in LM’s and FM’s relationships with hip aBMD, after adjustments for powerful covariates known to influence bone acquisition. In girls there was a trend of higher aBMD-levels across LM tertiles and FM tertiles at femoral neck and total hip. In boys, a positive trend was observed across LM tertiles at both sites. By contrast, across FM tertiles, such a trend was only present at the femoral neck site. In addition, low LM levels suggested a doubled likelihood for lower aBMD levels in boys compared to girls. Compared to FM, LM explained more of the variation in hip aBMD, in both genders. LM/FM-combinations including high LM stood out as most beneficial for aBMD, whereas in adolescents with low LM levels, FM was of considerable importance, observed more prominently in girls.

### Comparisons to other studies

In a cross-sectional study of Japanese adolescents in a comparable age group [30], Kouda et al. investigated relationships between bone variables and FM indices stratified by LM. In the lowest tertile of LM, FM index was significantly associated with aBMD_FN_ and whole body BMC for both genders, an association not seen in the other LM tertiles. Despite Kouda et al’s smaller study cohort (*n* = 235), the similarities in findings are striking, especially the association between FM and aBMD among adolescents with low LM. As summarized by Kouda et al., several studies of adolescents confirm the beneficial effect of LM on bone health, whereas studies on the associations between FM and bone provided conflicting results, some indicating a positive independent relationship [13, 14] while other indicate inverse relationships [14, 31–37]. The new important result from our study, beyond Kouda et al’s findings, is the observed gender variation. Another study from the present cohort revealed similar gender differences suggesting that relatively sedentary boys, with excess screen time had lower aBMD-levels compared to normal boys, whereas girls reporting such sedentary lifestyle had higher aBMD-levels than their more physical active peers [20]. Also Foley and colleagues in a longitudinal study of Tasmanian youths support this gender difference [38]. They followed girls and boys from pre-pubertal to late adolescence, and their study suggested that absolute LM and FM at pre-puberty played different roles at the age of 16. LM in boys predicted a positive change from tracking in aBMD at the hip and spine, whereas in girls the amount of FM not LM, had similar effect.

Our results are in agreement with previous findings in adults, like the meta-analysis on the association between LM, FM and aBMD [39], which included 4966 men and 15,260 women aged between 18 and 92 years. The correlation between LM and aBMD_FN_ was significantly higher than the correlation between FM and aBMD_FN_. As in our study the effect of LM was greater in men than in women. Also in a Norwegian cohort consisting of middle aged and elderly men and women, LM was more strongly correlated to aBMD_FN_ compared to FM [40]. Whereas FM was a significantly stronger predictor of aBMD in females than among men, particularly in the lower levels of LM, as described in our study.

### Mechanisms

With the strong association between LM and aBMD_FN_, Ho-Pham et al. concluded that physical activity is an important intervention for prevention of bone loss and osteoporosis in the adult population [39]. Our findings in adolescents support this notion. The mechanostat’s effect on bone [41] may explain the positive trend of physical activity and LM levels, which are associated with increasing aBMD-levels and with a strong influence of bone acquisition in youth. Peak momentary muscle forces are of importance of bone strength [41], in addition to loading effect of the muscle mass. Moreover, non-mechanical factors may modulate the mechanostat’s effect, and there is a growing understanding of how myokines released by muscles communicate with other organs including bone [42]. The probability for low bone density at lower LM levels, especially in boys, underscores the significance of LM during growth. And longitudinal results from this cohort; suggest that at least girls at 17 are close to PBM at the hip sites [43], indicating the need for early intervention to enhance bone mas levels. As indicated the LM amount is a reflection of physical activity levels, however bone and LM cannot be built without sufficient nutrition, such as energy, protein and calcium intake [44].

Several mechanisms contribute to the understanding of FM’s relationship with bone. Except greater load to the skeleton, secretions of adipocyte hormones (leptin) and pancreatic beta cell hormones (insulin etc.) are directly influenced by FM and associated with higher bone mass measures [45]. Moreover, high FM levels may be attributed to excess energy intake; along with glucose ingestion such a feeding effect may have an anabolic impact on bone (the gut-bone axis) [46]. In the FM – aBMD association the gender difference was more pronounced. In girls we found a trend of high aBMD-levels across FM tertiles at both hip sites, while in boys only at the femoral neck site. According to Green and Naughthon the femoral neck is highly exposed to weight [47], which includes both genders, whereas the effect of FM at total hip in girls suggests other explanations, such as hormonal implications.

An important implication of the findings from our study is that modifiable lifestyle factors seem to have a strong influence on peak bone mass attainment, and possible later fracture risk. The aBMD_FN_ differences between the low LM/FM- and the high LM/FM-combinations were 0.129 g/cm^2^ and 0.149 g/cm^2^ in girls and boys respectively. These numbers equals one SD aBMD in this study population close to PBM level and correspond to a fracture risk difference of 50% in adulthood [4].

### Strengths and weaknesses

The strength of this population-based study is connected to the large representative sample of Norwegian adolescents, quite homogenous according to age and ethnicity. The survey setup ensured high quality data acquisition, allowing control for important confounding factors. The results are in line with previous findings, and the large sample size allows sub-group analyses. Due to incomplete data 15% girls and 26% boys younger than 18 were excluded from analyses, mainly because of missing vitamin D-levels (girls) and puberty score (boys). The excluded girls were shorter, with higher BMI and FM levels. While the excluded boys were slightly younger, at an earlier stage of puberty, reported lower physical activity levels and more frequent smoking. It is not likely an inclusion of those participants would have altered the conclusions, but it may have strengthened the findings in girls and attenuated the boys’ results.

The main limitation is the cross sectional design, which capture only a temporal window of young adolescent life. In this age-group rapid changes in height, weight and pubertal status may still occur. The inconsistency in conclusions of FM’s relationship with bone in adolescents may be a result of a delicate and changing relationship during age and hormonal state [7]. Unfortunately, beyond the self-reported menarche age and PDS-score we had no information on hormonal levels, which may have influenced the results. Information on the distribution between visceral fat and subcutaneous fat might as well have shed light on the FM–bone relationship in this age group, as the fat-bone relationship may depend on site-specific fat depositions rather than total body fat mass [17].

The main outcome, aBMD derived by DXA-scans has limitations connected to being two-dimensional, rather than three-dimensional. By not take into account the third dimension, the technique is unable to measure the true volumetric density [48]. However, the DXA measurement at the hip is reliable and widely used [49], and the DXA approach is the preferred method for clinical measurements in children and adolescents who have not reached peak bone mass. Due to difficulties in identifying bony landmarks in the hip region, measurements of the spine or total body less head are regarded more reliable and recommended for children and adolescents [48]. In this study estimates at the spine were not available. Anyhow, the research technicians were well trained, which will reduce the inter-observer variability and random error. Even though additional information from measurements at the spine would have contributed to better insight, it is not likely that it would have changed the conclusions.

To our knowledge in addition to the study of Foley and colleagues, two longitudinal studies in corresponding age groups have been published, both on structural analyses of bone strength. Jackowski et al. investigated LM’s influence on bone during adolescence into early adulthood, and concluded that LM contributes to structural strength at the hip in both genders [50], while Streeter et al. concluded, based on 7 years of follow-up, that body fat is not deleterious for bone quality in pre- and peripubertal adolescents [51]. They suggested that in girls body fat was related to larger and stronger bones, whereas in boys only to bone strength. A follow-up study of our cohort will provide information on both components of body composition’s impact during further growth.

## Conclusions

This cross sectional study among 15–17 year old Norwegian adolescents, supports the importance of LM as a strong independent predictor for bone mass, along with FM’s significant contribution to bone mass in girls and in adolescents with lower lean mass levels. Our observations indicate a gender specific variation; high lean mass levels were strongly associated with boys’ aBMD-levels at the hip, which highlights the negative relationship between a sedentary lifestyle and bone strength. In contrast, the results indicated a more complex balance between lean mass and fat mass in adolescent girls. The gender specific delicate balance between fat and lean mass pin points the significance of nutrition and physical activity for sound bone health in adolescence.

## Abbreviations

aBMD: Areal bone mineral density
aBMD_FN_: aBMD femoral neck
aBMD_TH_: aBMD total hip
ANCOVA: analysis of covariance
BMI: Body Mass Index
CI: Confidence interval
CV: Coefficient of variation
DXA: Dual-energy x-ray absorptiometry
FM: Fat mass
LM: Lean mass
OR: Odds ratio
PDS: Pubertal Development Scale
SD: Standard deviation
SPSS: Statistical Package for the Social Sciences
TFF1: The Tromsø Study, Fit Futures 1
UiT: UiT The Arctic University of Norway
UNN HF: University Hospital of North Norway

## References

[1] Marshall D, Johnell O, Wedel H (1996). Meta-analysis of how well measures of bone mineral density predict occurrence of osteoporotic fractures. BMJ (Clin Res ed).

[2] Cooper C, Westlake S, Harvey N, Javaid K, Dennison E, Hanson M (2006). Review: developmental origins of osteoporotic fracture. Osteoporos Int.

[3] Baxter-Jones AD, Faulkner RA, Forwood MR, Mirwald RL, Bailey DA (2011). Bone mineral accrual from 8 to 30 years of age: an estimation of peak bone mass. J Bone Miner Res.

[4] Rizzoli R, Bianchi ML, Garabedian M, McKay HA, Moreno LA (2010). Maximizing bone mineral mass gain during growth for the prevention of fractures in the adolescents and the elderly. Bone.

[5] Heaney RP, Abrams S, Dawson-Hughes B, Looker A, Marcus R, Matkovic V, Weaver C (2000). Peak bone mass. Osteoporos Int.

[6] Kanis JA, Borgstrom F, De Laet C, Johansson H, Johnell O, Jonsson B, Oden A, Zethraeus N, Pfleger B, Khaltaev N (2005). Assessment of fracture risk. Osteoporos Int.

[7] Reid IR (2010). Fat and bone. Arch Biochem Biophys.

[8] De Laet C, Kanis JA, Oden A, Johanson H, Johnell O, Delmas P, Eisman JA, Kroger H, Fujiwara S, Garnero P (2005). Body mass index as a predictor of fracture risk: a meta-analysis. Osteoporos Int.

[9] Lee SH, Desai SS, Shetty G, Song HR, Lee SH, Hur CY, Lee JC (2007). Bone mineral density of proximal femur and spine in Korean children between 2 and 18 years of age. J Bone Miner Metab.

[10] Winther A, Dennison E, Ahmed LA, Furberg AS, Grimnes G, Jorde R, Gjesdal CG, Emaus N (2014). The Tromso study: fit futures: a study of Norwegian adolescents' lifestyle and bone health. Arch Osteoporos.

[11] Travison TG, Araujo AB, Esche GR, JB MK (2008). The relationship between body composition and bone mineral content: threshold effects in a racially and ethnically diverse group of men. Osteoporos Int.

[12] Zhu K, Hunter M, James A, Lim EM, Walsh JP (2015). Associations between body mass index, lean and fat body mass and bone mineral density in middle-aged Australians: the Busselton healthy ageing study. Bone.

[13] Pietrobelli A, Faith MS, Wang J, Brambilla P, Chiumello G, Heymsfield SB (2002). Association of lean tissue and fat mass with bone mineral content in children and adolescents. Obes Res.

[14] Ackerman A, Thornton JC, Wang J, Pierson RN, Horlick M (2006). Sex difference in the effect of puberty on the relationship between fat mass and bone mass in 926 healthy subjects, 6 to 18 years old. Obesity (Silver Spring).

[15] Arabi A, Nabulsi M, Maalouf J, Choucair M, Khalife H, Vieth R, El-Hajj Fuleihan G (2004). Bone mineral density by age, gender, pubertal stages, and socioeconomic status in healthy Lebanese children and adolescents. Bone.

[16] Ausili E, Rigante D, Salvaggio E, Focarelli B, Rendeli C, Ansuini V, Paolucci V, Triarico S, Martini L, Caradonna P (2012). Determinants of bone mineral density, bone mineral content, and body composition in a cohort of healthy children: influence of sex, age, puberty, and physical activity. Rheumatol Int.

[17] Dimitri P, Bishop N, Walsh JS, Eastell R (2012). Obesity is a risk factor for fracture in children but is protective against fracture in adults: a paradox. Bone.

[18] Weaver CM, Gordon CM, Janz KF, Kalkwarf HJ, Lappe JM, Lewis R, O'Karma M, Wallace TC, Zemel BS (2016). The National Osteoporosis Foundation's position statement on peak bone mass development and lifestyle factors: a systematic review and implementation recommendations. Osteoporos Int.

[19] Farr JN, Dimitri P (2017). The impact of fat and obesity on bone microarchitecture and strength in children. Calcif Tissue Int.

[20] Winther A, Ahmed LA, Furberg A-S, Grimnes G, Jorde R, Nilsen OA, Dennison E, Emaus N (2015). Leisure time computer use and adolescent bone health—findings from the Tromsø study, fit futures: a cross-sectional study. BMJ Open.

[21] The Tromsø Study [http://www.tromsostudy.com] Accessed 22 June 2015.

[22] Lunar enCore, Supplement til pediatrisk referansedata, 1. revision edn: GE Healthcare; 2010.

[23] Omsland TK, Emaus N, Gjesdal CG, Falch JA, Tell GS, Forsen L, Berntsen GK, Meyer HE (2008). In vivo and in vitro comparison of densitometers in the NOREPOS study. J Clin Densitom.

[24] Oberg J, Jorde R, Almas B, Emaus N, Grimnes G (2014). Vitamin D deficiency and lifestyle risk factors in a Norwegian adolescent population. Scand J Public Health.

[25] Saltin B, Grimby G (1968). Physiological analysis of middle-aged and old former athletes. Comparison with still active athletes of the same ages. Circulation.

[26] Petersen A, Crockett L, Richards M, Boxer A (1988). A self-report measure of pubertal status: reliability, validity, and initial norms. J Youth Adolescence.

[27] Nordic Nutrition Recommendation 2012. Part 5 [http://norden.diva-portal.org/smash/record.jsf?pid=diva2%3A745817&dswid=8635] Accessed 9 Feb 2015.

[28] Wang Q, Seeman E. Skeletal Growth and Peak Bone Strength. In: Primer of the Metabolic Bone Diseases and Disorders of Mineral Metabolism. Eight edn. Edited by Rosen CJ. Iowa, USA: Wiley; 2013:127–34.

[29] Kirkwood BR, Sterne JA. Essential Medical Statistics, Second Edition edn. Oxford, UK: Blackwell Science LTD; 2003.

[30] Kouda K, Fujita Y, Sato Y, Ohara K, Nakamura H, Uenishi K, Iki M (2014). Fat mass is positively associated with bone mass in relatively thin adolescents: data from the Kitakata kids health study. Bone.

[31] Garnett SP, Hogler W, Blades B, Baur LA, Peat J, Lee J, Cowell CT (2004). Relation between hormones and body composition, including bone, in prepubertal children. Am J Clin Nutr.

[32] Clark EM, Ness AR, Tobias JH (2006). Adipose tissue stimulates bone growth in prepubertal children. J Clin Endocrinol Metab.

[33] El Hage RP, Courteix D, Benhamou CL, Jacob C, Jaffre C (2009). Relative importance of lean and fat mass on bone mineral density in a group of adolescent girls and boys. Eur J Appl Physiol.

[34] Weiler HA, Janzen L, Green K, Grabowski J, Seshia MM, Yuen KC (2000). Percent body fat and bone mass in healthy Canadian females 10 to 19 years of age. Bone.

[35] Lazcano-Ponce E, Tamayo J, Cruz-Valdez A, Diaz R, Hernandez B, Del Cueto R, Hernandez-Avila M (2003). Peak bone mineral area density and determinants among females aged 9 to 24 years in Mexico. Osteoporos Int.

[36] Janicka A, Wren TA, Sanchez MM, Dorey F, Kim PS, Mittelman SD, Gilsanz V (2007). Fat mass is not beneficial to bone in adolescents and young adults. J Clin Endocrinol Metab.

[37] Mosca LN, da Silva VN, Goldberg TB (2013). Does excess weight interfere with bone mass accumulation during adolescence?. Nutrients.

[38] Foley S, Quinn S, Jones G (2009). Tracking of bone mass from childhood to adolescence and factors that predict deviation from tracking. Bone.

[39] Ho-Pham LT, Nguyen UD, Nguyen TV (2014). Association between lean mass, fat mass, and bone mineral density: a meta-analysis. J Clin Endocrinol Metab.

[40] Gjesdal CG, Halse JI, Eide GE, Brun JG, Tell GS (2008). Impact of lean mass and fat mass on bone mineral density: the Hordaland health study. Maturitas.

[41] Frost HM, Schonau E (2000). The "muscle-bone unit" in children and adolescents: a 2000 overview. J Pediatr Endocrinol Metab.

[42] Pedersen BK (2013). Muscle as a secretory organ. Compr Physiol.

[43] Nilsen OA, Ahmed LA, Winther A, Christoffersen T, Furberg AS, Grimnes G, Dennison E, Emaus N (2017). Changes and tracking of bone mineral density in late adolescence: the Tromso study, fit futures. Arch Osteoporos.

[44] Bass SL, Eser P, Daly R (2005). The effect of exercise and nutrition on the mechanostat. J Musculoskelet Neuronal Interact.

[45] Reid IR (2008). Relationships between fat and bone. Osteoporos Int.

[46] Yavropoulou MP, Yovos JG (2013). Incretins and bone: evolving concepts in nutrient-dependent regulation of bone turnover. Hormones (Athens).

[47] Greene DA, Naughton GA (2006). Adaptive skeletal responses to mechanical loading during adolescence. Sports Med.

[48] Blake G, Adams JE, Bishop N. DXA in Adults and Children. In: Primer of the Metabolic Bone Diseases and Disorders of Mineral Metabolism. Eight edn. Edited by Rosen CJ. Iowa, USA: Wiley; 2013:251–63.

[49] Cummings SR, Bates D, Black DM (2002). Clinical use of bone densitometry: scientific review. JAMA.

[50] Jackowski SA, Lanovaz JL, Van Oort C, Baxter-Jones AD (2014). Does lean tissue mass accrual during adolescence influence bone structural strength at the proximal femur in young adulthood?. Osteoporos Int.

[51] Streeter AJ, Hosking J, Metcalf BS, Jeffery AN, Voss LD, Wilkin TJ (2013). Body fat in children does not adversely influence bone development: a 7-year longitudinal study (EarlyBird 18). Pediatric obesity.

